# Supplementary material for: Assessment of the Chad guinea worm surveillance information system: A pivotal foundation for eradication
Source: PLoS Negl Trop Dis. 2021 Aug 9;15(8):e0009675. doi: 10.1371/journal.pntd.0009675 (PMC8376011; doi:10.1371/journal.pntd.0009675)
Supplement: Attachment — Submitted filename: ResponseLetter_InformaticsAssessmentGW_Final.docx [file pntd.0009675.s001.docx]

June 21, 2021.

Dr. Zvi Bentwich,

Associate Editor

PLoS Neglected Tropical Diseases

Dr. Achim Hoerauf

Deputy Editor

PLoS Neglected Tropical Diseases

Saugat Karki, MD, MS

Public Health Informatics Fellow

Centers for Disease Control and Prevention

[SKarki@cdc.gov](mailto:SKarki@cdc.gov)

Dear Bentwich, Dr. Hoerauf, and members of the editorial team,

Thank you for the opportunity to revise our manuscript entitled ‘Assessment of the Chad Guinea Worm Surveillance Information System: A Pivotal Foundation for Eradication’. We appreciate the careful review and constructive suggestions. We have adopted the suggestions and addressed all of the reviewers’ comments, and believe that the manuscript is substantially improved after making the suggested edits.

Following this letter are the reviewer comments in bold, with our responses in italics, and excerpts copied from the manuscript in ‘roman’ (not italicized). Specifically, we have added additional background on previous studies and the gap that our study addresses, and significantly revised and restructured the Methods section. As suggested, we have also added a table in the Discussion section that lists key issues identified in the assessment (and listed in the results section) and explains how these were addressed in the proposed road map. We have generalized this information in a way that could be replicated by other programs.

We have also uploaded a version of the manuscript with tracked changes denoting the changes made along with a clean version of the manuscript.

Thank you, again, for your consideration of our submission.

Sincerely,

**Key Review Criteria Required for Acceptance?
As you describe the new analyses required for acceptance, please consider the following:
Methods
-Are the objectives of the study clearly articulated with a clear testable hypothesis stated?
-Is the study design appropriate to address the stated objectives?
-Is the population clearly described and appropriate for the hypothesis being tested?
-Is the sample size sufficient to ensure adequate power to address the hypothesis being tested?
-Were correct statistical analysis used to support conclusions?
-Are there concerns about ethical or regulatory requirements being met?
Reviewer #1: The methods are structured less than effectively. Two of the earlier sections (Initial description, Mental models) might perhaps better placed in the Introduction, as they describe the starting place. That said, this section also would be hard to understand for someone who was not already familiar with the GW eradication program or very similar efforts. A further step back is required to understand the structures and practices of the program, and particularly how these use, or do not use, data collection and analysis.**

*In response to the reviewer’s comment, we have moved the contents of the ‘Initial description’ subsection to the Introduction section. We now begin the Methods section with an ‘Ethics Statement’, followed by the description of how information was collected for this study in the ‘Information Gathering and Data Quality Interviews’ subsection. We further edited the content in the methods section as advised by the reviewers (addressed in detail below). We summarized the methods used in this study in the last two paragraphs by adding the following text in the body of the manuscript:* “We mapped the concepts identified in the IVC to the DISC domains to create a contextual analysis matrix. This IVC-DISC framework has been frequently used to guide the solutions for various informatics problems in public health [28, 29]. Concepts identified on the IVC were further categorized into granular contexts of the DISC framework. This IVC-DISC contextual analysis matrix reveals each concept identified in the IVC in the context of data, technology, people and process, organization, and environment. It is an evaluation model for assessing information systems, the value that they create, and their data flow. We used IVC-DISC framework as it breaks down contributors of the information system which are otherwise difficult to isolate. This allows for a holistic examination of these contributors and at the same time also allows for a measurable and quantifiable elicitation of these contributors and its role in the system as a whole.

We reviewed data collection, reporting, and management practices to map data elements and document workflows to provide insight into the data quality and identify areas for process improvement. IVC and DISC frameworks brough out the strengths and weaknesses of the information system and laid the foundation for streamlined processes.”

*Regarding the need to explain the structures and practices of the CGWEP, we have added some text to the Introduction to briefly explain the existing information system. We also refer to previously published works that describe the surveillance system in great detail and utilize data yielded from the system. We added the following text to the Introduction section in the manuscript: “*

At the time of this investigation, Guinea worm surveillance data were collected in a siloed manner with inefficiencies in data management processes. For example, information collected regarding the same Guinea worm case was not linked because data were collected in isolation via paper-based surveys. Although the existing information system worked well in the past, as evidenced by the 99.9% reduction in human Guinea worm cases since the mid-1980s [1,15, 16], in the context of eradication, identifying and containing every single case becomes increasingly difficult as cases decline [17, 18]. At the same time, the number of Guinea worm “rumors” (unverified reports of possible cases) in Chad has increased substantially. For example, Guagliardo et al (2020) reported that there were 504 dog rumors in 2015 compared to 15,511 rumors in 2018 [4]. The enormous increase in rumors over time has underscored the need for improved information systems to facilitate detection, tracking, and containment.” *and* “Although a previous, rigorous surveillance evaluation had been conducted [20], it was determined that an additional assessment would be needed specifically to address informatics needs.”

*To make it clearer, we further summarized this by adding a ‘Surveillance System’ subsection in the Methods section:* “CGWEP is run by the Chad MoPH with support from The Carter Center and operates in approximately 2,300 villages throughout Southern Chad. Surveillance is simultaneously conducted in both humans and domestic animals (primarily dogs) [22], and involves both active and passive surveillance, described in detail elsewhere [4]. The data collected by the surveillance system are owned by the government of Chad and have been used previously to discern epidemiologic trends in humans and dogs alike [4, 23].”

*To take a step back and explain how these data are used, we added the following to the Introduction:* “The Chad Guinea Worm Eradication Program (CGWEP) uses data to inform these prevention and control strategies, and to create periodic and ad hoc reports that are disseminated to partners and stakeholders.” **While the Methods provide an outline of the approach, they are less clear on the analytical process.**

*We added a subsection (‘Information Gathering and Data Quality Interviews’) in the Methods section to describe how information was collected. Additional changes to clarify the analytical process in the Methods and Results sections include:*

- *Moved the summary of the process of how we arrived at the results from the first paragraph of the results to the last paragraph of the methods section. This should further help tie together the methods and the analytical process. We also added the following text to summarize the methods as the last paragraph of the Methods section and emphasize the analytical process: “*We reviewed data collection, reporting, and management practices to map data elements and document workflows, to provide insight into the data quality, and to identify areas for process improvement. IVC and DISC frameworks brought out the strengths and weaknesses of the information system and laid the foundation for streamlined processes”
- *We summarized the ‘Information Gathering and Data Quality Interviews’ subsection that further explained the analytical process by adding the following:* “Findings from the interviews informed the development of mental models (described below) to reveal strengths and weaknesses of these processes.”
- *We added a summary of the ‘Mental Models’ subsection and outlined the step-by-step process how the mental models were filled in with concepts identified and how the IVC-DISC framework was organized. We further outlined the analytical outcome of this process:* “We mapped the concepts identified in the IVC to the DISC domains to create a contextual analysis matrix. This IVC-DISC framework has been frequently used to guide the solutions for various informatics problems in public health [28, 29]. Concepts identified using the IVC were further categorized into granular contexts using the DISC framework. This IVC-DISC contextual analysis matrix reveals each concept identified in the IVC in the context of data, technology, people and process, organization, and environment. It is an evaluation model for assessing information systems, the value that they create, and their data flow. We used IVC-DISC framework as it breaks down contributors to the information system that are otherwise difficult to isolate. This allows for a holistic examination of these contributors and at the same time also allows for a measurable and quantifiable elicitation of these contributors and their roles in the system as a whole.”
- *We also modified the following text in the ‘Mental Models’ subsection in the Results section that specified an example of the analytical outcome of the IVC-DISC matrix:* “For example, concepts of data capture identified in the IVC were mapped to the DISC framework. This included the type of information collected; the technology used for data collection; the people and processes collecting the data; the organizational structure for data collection and use from the village-level to the international level; and the environmental factors affecting these processes.”

**Respondents: While the respondents were asked for consent, was informed consent given, and was it verbal or written? Which authority granted ethical approval for the study? What form did the consultation take? Face-to-face interviews? How many respondents of each type were there? This is important to understand how the practices were sampled. Some of this information is provided in a Supplementary Table, but this does not appear to be cited in the paper text, and so comes as a surprise at the end. This Table could be included in the main paper.**

*Consent was verbal, interviews were face-to-face, and CDC gave the formal ethical approval for the study. We have added an ‘Ethics Statement’ subsection to the Methods to specify this:* “All respondents were asked for verbal consent before proceeding with the interviews about data quality. This project was given a non-research determination by the delegated authority at the Center for Global Health, Centers for Disease Control and Prevention (protocol #: 0900f3eb819fb155).”

*As the reviewer requests, we have also moved Table 1 to the main text.* **This Table suggests that there is a heavy bias in sampling towards fairly senior, and office-based, staff. The most "junior" are data entry staff and Technical Advisors, also all based in the Chad country office. An important point, therefore, is that no true "field staff" i.e. those who actually collect the data appear to have been sampled. Which seems like a serious omission in terms of understanding data generation and subsequent flows.**

*The reviewer does point out a limitation in our methods in that the majority of the information we collected was from office staff rather than field staff. Our rationale for this was that the office staff have more of a role in managing and maintaining the information system itself – but of course the field staff do have a very important role in data collection. Relatedly, our group recently published a surveillance evaluation (Rubenstein et al, 2021), which did involve extensive questioning of field staff, though the focus of that project was more on data collection rather than on informatics.*

*We have added this as a limitation in the Discussion section:* “Field staff that are responsible for data collection were not interviewed for this project, as we instead focused our information gathering on GCWEP and Carter Center senior-level office staff based in N’Djamena and Atlanta (Table 1). This could bias our findings by omitting important weaknesses in the information system only visible to GCWEP members that collect data on the ground.”

*As suggested, we moved table describing the respondents to the main paper. Here, we replaced ‘Country Office’ with ‘CGWEP’. ‘Country Office’ may have inadvertently represented Country Headquarters; it was instead used to differentiate Chad program in general from the Atlanta headquarters.*  **Reviewer #2: This paper is very weak on identifying related work, research questions and research methods. see below**

*We have attempted to improve this work by adding a summary of related work to the introduction. Some previous informatics work related to NTDs added are:*

- *A viewpoint outlining the use of technology for NTDs [8]*
- *An assessment of paper-based and digital tools of data collection in low to middle income countries, specifically as it pertains to data quality [10]*
- *A pilot study comparing electronic data collection and paper-based questionnaires for trachoma cases in Ethiopia [11]*
- *A review of digital health technologies used in neglected tropical diseases [12]*
- *A report describing an SMS based tool for assessing lymphatic filariasis in Malawi and Ghana [13]*
- *A systematic review of innovative tools used by mobile health strategies to tackle skin NTDs [14]*

*Previous work specific to Guinea worm disease in Chad cited in the manuscript:*

- *Summary of eradication efforts – 2017-2018 and 2018-2019 [1, 16]*
- *Description of CGWEP surveillance system and analyses of the data from the surveillance system of CGWEP [4, 20, 23]*
- *Epidemiology of Guinea worm in Chad [5, 6]*

*Previous work related to Guinea worm eradication efforts cited in the manuscript:*

- *Cost-effectiveness [17]*
- *Challenges [18]*

*We also recognize that this manuscript is unconventional in that we are not really testing a hypothesis or evaluating research questions per se. As mentioned above, there are limited informatics or data management framework-related papers and this manuscript attempts to fill that gap in literature. Therefore, we felt it was important information to share with the broader NTD community, as many NTD programs are shifting from paper-based data collection to electronic data capture. We have attempted to clarify this in the last paragraph of the Introduction section (Objective subsection):* “The purpose of this report is to describe the approach and methods used to in the Info-Aid, and to outline a roadmap to improve CGWEP information systems’ data flow, upstream and downstream data management practices, and system inefficiencies. Previously published studies revealed a gap in a systems-level assessment of information systems unique to NTDs [10], and therefore, this approach could also be adopted by other NTD programs [21].”

*We have significantly re-organized the Methods section to add more clarity. This can be found throughout the address of reviewers’ comments here and in the body of the manuscript.* **Reviewer #3: Partially. This paper is a report of a mental map to evaluate the data collection processes of the CGWEP. There are no real data included and no statistical analyses.**

*As we note above, we acknowledge that this is an unconventional paper, with no data or statistical analyses. However, there are limited informatics or data management framework-related papers and this manuscript attempts to fill that gap in literature.*  **--------------------
Results
-Does the analysis presented match the analysis plan?
-Are the results clearly and completely presented?
-Are the figures (Tables, Images) of sufficient quality for clarity?
Reviewer #1: Table 1 feels more like Results than Methods. Are forms 10 and 11 the same, as their description differs in only 1 word "Systematic"? Perhaps their function/content could be distinguished better?**

*Thank you for highlighting this discrepancy regarding these forms. Forms 10 and 11 are two different versions of the same form, with form 10 being the 2018 version and 11 being the 2017 version. There does not seem to be a justification to keep them both and removed the 2017 version. This form is used by field staff as a worksheet to calculate the volume of a water source and the amount of Abate to be applied. It is used for a single water source and is a record of the raw data used for calculations and not so much for monitoring.*

*Form 12 is a summary form of all the abate treatments that took place during the month in a particular coverage area and is not actually for determining calculations. Field staff simply transfer the final calculations from each of the form 10 onto Form 12 as a summary at the end of the month.*

*Below are the adjustments to the language in the table that might help further clarify.*

| Form number | Name of survey form | Brief description |
| --- | --- | --- |
| 10 | Abate^®^ (temephos) application form | Programmatic use |
| 11 | Summary form of Abate^®^ (temephos) treatment | Programmatic use |

*Reviewer #1 suggests that table 2 (previously table 1) be moved to the Results section. However, we are merely listing what exists in the program and demonstrating a source of our information for the data collection and mapping exercise. For these reasons, we do not think that the table is suitable for the Results section.*  **I am actually surprised there are as few as 15 forms, which suggests a good deal of additional ad hoc data collection and distribution that covers processes such as collection of worms for despatch for identification, counts of animals during censuses, documentation of village visits by a range of voluntary and paid staff, and other programmatic activities.**

*It is true that there are additional forms for some of these program-oriented activities. We tried to focus this effort on the forms related to surveillance about cases in humans and animals, therefore excluding forms related to more logistical processes. We have added the following text to the Methods to explain this:* “The program may use ad hoc processes for collecting data related to the logistical processes. However, we focused on the surveillance forms as these are routine processes and are the source of all the data related to Guinea worm epidemiology and eradication activities.” **Because of the lack of detail in the Methods, the first part of the results are a surprise as they deal with very specific challenges to data collection and recording (not recording dog containment), rather than the information systems. This elicitation of problems is not addressed in any detail in the Methods, which deal primarily with structural aspects. This specific aspect (dog containment) is then discussed in some detail, which again seems out of place here. Were no other aspects of the program considered equally challenging?**

*We have changed the methods section significantly to address the Reviewer’s concerns. We think that the edited version better explains the methods used that revealed the outcomes described in the manuscript.*

*In the ‘Data Quality’ subsection of the Results section, we discuss data collection challenges in particular with the quality of data. We use data related to canine containment practice as an example how there are challenges at the source which might affect the quality of the data. We have completely changed this section to briefly use canine containment data as an example only and then generalize challenges with data quality at the data collection level. We modified the text in the manuscript as follows: “*Issues related to data quality were expressed by the interviewees. For example, community members (and potentially even field staff) may be inaccurately reporting data, especially canine containment data. The standard for containment requires people to tether their dogs at the blister stage, before worm emergence, but supervisors usually cannot arrive to the field location in time to verify that the dog was indeed tethered at the blister stage. Visible blisters must be promptly recognized so that dogs are quickly contained. Timing is critical for containment because there are often only a few hours between development of a blister and worm emergence, when the dog will contaminate water sources. A further challenge to surveillance is that Guinea worms in dogs are difficult to identify, especially before the worms have emerged, as blisters often occur near the paws or in between toes and are obscured by fur. A further challenge to surveillance is that Guinea worms in dogs are difficult to identify, especially before the worms have emerged, as blisters often occur near the paws or in between toes and are obscured by fur.”

*This addition of generalizing data quality issues was missing in our previous submitted version, thank you for highlighting this, we hope this alleviates this concern. The following text was added to generalize the discussion, and remove the inadvertent focus on canine data present in the previously submitted version:* “Apart from these containment challenges with respect to timing and identification, paper-based data collection also jeopardized the quality of surveillance. Paper-based survey forms are prone to physical damage or loss and errors in handwritten information is a reality. Data could be inadvertently or maliciously changed, and auditing is more difficult because of the lack of version control.” **There follow no further results relating to data content or quality, which again is surprising. It does not feel as though the data requirements, for actual control, are at all well explored with the respondents. Instead, the analysis is largely based on the forms and their overlapping content and functions. Are the authors therefore content that all the necessary information is being collected and that the only improvement is in how these data are handled?**

*Thank you for raising this issue. This is an important issue and it was not our intent to minimize discussion of its importance in the evaluation. To address the Reviewer’s comment, we significantly rearranged the methods section and added a subsection (‘Information Gathering and Data Quality Interviews’) that summarizes how the results were obtained. The text added in this subsection is as follows:*

“We gathered relevant literature and documentation regarding the informatics landscape and CGWEP operations. Although there were data dictionaries for some surveys, no program-level metadata and data dictionary existed.

To document the public health context, existing information systems (people, process, and technology), and informatics need and gaps, we consulted with staff from CGWEP and The Carter Center in N’Djamena and Atlanta during August and September of 2019 (Table 1). Two interviews were conducted in French with the aid of an interpreter and the remaining interviews were conducted in English. Interviews were guided by semi-structured scripts that were tailored to a range of respondent roles (i.e., MoPH representatives, data managers, field staff, The Carter Center leadership). Questions probed about the simplicity of the surveillance system and the quality of the data [24]. For example, we requested explanations about how information is transferred at and between different levels of the system (i.e., village to field supervisors to regional hub to national level), with special attention to any potential bottlenecks. We asked if there were certain aspects of the surveillance system that repeatedly stimulate complaints from workers, require repeated training to maintain competency or required adjustments to problem solve. We also asked if information was captured at a specific-enough level of detail to support needed analyses, and if government officials, community leaders, and the general public trust the quality of the data for programmatic decisions. Findings from the interviews informed the development of mental models (described below) to reveal strengths and weaknesses of these processes.”

*As suggested by reviewer #1, we have moved the supplementary table (now Table 1) to the main body to report what type of information was collected from whom. This table reveals, the type of issues examined, and the information obtained during those discussions, which are well beyond just examining the survey forms and the overlap of content contained in them. We further created a new table (Table 4 [please find in the body of the manuscript submitted with this letter]) that highlights the outcome and the key issues discovered in our assessment. The IVC-DISC framework assessed the information system from a holistic perspective, we added this detail in the manuscript: “*We mapped the concepts identified in the IVC to the DISC domains to create a contextual analysis matrix. This IVC-DISC framework has been frequently used to guide the solutions for various informatics problems in public health [28, 29]. Concepts identified using the IVC were further categorized into granular contexts using the DISC framework. This IVC-DISC contextual analysis matrix reveals each concept identified in the IVC in the context of data, technology, people and process, organization, and environment. It is an evaluation model for assessing information systems, the value that they create, and their data flow. We used IVC-DISC framework as it breaks down contributors to the information system that are otherwise difficult to isolate. This allows for a holistic examination of these contributors and at the same time also allows for a measurable and quantifiable elicitation of these contributors and their roles in the system as a whole.

We reviewed data collection, reporting, and management practices to map data elements and document workflows, to provide insight into the data quality, and to identify areas for process improvement. IVC and DISC frameworks brought out the strengths and weaknesses of the information system and laid the foundation for streamlined processes.”

*As such our assessment is more comprehensive than just based on the forms and their overlapping content and functions*.

*For this study, we focused on the informatics aspects of CGWEP, related to how the information already collected was managed and how it could be improved. Relatedly, our group recently published a surveillance evaluation (Rubenstein et al, 2021), which involved the study of the surveillance system and data collection. This published study explored whether the data collected was adequate or not.*  **Similarly, surveillance sensitivity (L163) is not described specifically. And is not, in itself directly related to informatics processes, although it is certainly of importance.**

*Thank you for highlighting the ambiguity that the brief discussion of surveillance sensitivity caused. Surveillance sensitivity may not be best discussed in this manuscript as this manuscript describes the informatics and information collection, management, and use aspects of the surveillance system. Surveillance sensitivity is discussed in detail elsewhere in another publication, so we removed this from here and instead referenced a recently-published surveillance evaluation (Rubenstein et al, 2021).* **L188-199 are again Methodological in tone and content, while the lines 193-197 are not easily understood and their purpose is not clear. it is not clear how this analytical outcome arises from the practical work undertaken. This highlights the missing content that would allow these components to be joined in the mind of the reader.**

*Thank you for bringing up the concern that our previous description lacked some clarity and detail. To address the reviewer’s concern that analytical outcome are not clear from the practical work undertaken, we significantly revised the methods section. We also added a subsection ‘Information Gathering and Data Quality Interviews’ that illustrates how we gathered this information, and specifically added language in the methods section to tie together how we arrived at the outcome from the practical work undertaken (*“For example, we requested explanations about how information is transferred at and between different levels of the system (i.e., village to field supervisors to regional hub to national level), with special attention to any potential bottlenecks. We asked if there were certain aspects of the surveillance system that repeatedly stimulate complaints from workers, require repeated training to maintain competency or required adjustments to problem solve. We also asked if information was captured at a specific-enough level of detail to support needed analyses, and if government officials, community leaders, and the general public trust the quality of the data for programmatic decisions. Findings from the interviews informed the development of mental models (described below) to reveal strengths and weaknesses of these processes.”). *Lines 193-197 of the first submission were further broken down, parts of it detailing the method were moved to the method section and only an example of the how the IVC-DISC framework created analytical value is explained here.*

*We further modified the tone and content in this section to better reflect a ‘Result’ section. We moved the first paragraph of the results to the methods section, as it was a summary of the process of how we arrived at our results. We removed the sentence language that might contribute to some ambiguity (“This IVC-DISC contextual …. From the information context as”) and specifically explained that how concepts in the IVC figure were mapped to DISC framework by demonstrating an example of this process:* “For example, concepts of data capture identified in the IVC were mapped to the DISC framework. This included the type of information collected; the technology used for data collection; the people and processes collecting the data; the organizational structure for data collection and use from the village-level to the international level; and the environmental factors affecting these processes. Similarly, this was done for all other functions of the information system. Contextualizing components of information systems quantified measurable potential changes that can be introduced to streamline the process and improve efficiency.” *Hopefully these ties in the methods to the results and makes the practical work undertaken and the analytical outcomes arisen apparent.*  **L213-214. This is the first mention of the use of program data in epidemiological or other research activities. This might be expanded and made more prominent throughout the paper, as this is an additional vital use of program information.**

*Thank you for this suggestion. We have added an explanation of how data is used in the introduction section, public health context subsection:* “The Chad Guinea Worm Eradication Program (CGWEP) uses data to inform these prevention and control strategies, and to create periodic and ad hoc reports that are disseminated to partners and stakeholders.” **Reviewer #2: see below
Reviewer #3: Yes
--------------------
Conclusions
-Are the conclusions supported by the data presented?
-Are the limitations of analysis clearly described?
-Do the authors discuss how these data can be helpful to advance our understanding of the topic under study?
-Is public health relevance addressed?
Reviewer #1: While the conclusions are valuable and supported by the study, there is little express consideration of the constraints acting upon the roadmap, which are likely of considerable general relevance to NTD control programs. How might this roadmap be implemented in a timely way in the circumstances found in Chad, and other similar settings?**

*Thank you for raising this important concern. To address this concern, we have created a new table (Table 4 [please find in the body of the manuscript submitted with this letter]), that specifically details the considerations and constraints for implementation. Additionally, to acknowledge the constraints, we have added some explanation to the limitation subsection in the discussion section of the manuscript:* “The phased plan we propose here may not be suitable for all NTD programs because of the financial and human resources required and because of connectivity issues in remote areas that may hinder the ability to transmit and receive data. In the process of this transition CGWEP did incur costs related to monthly data transfer and storage, the cost of purchasing 700 cell phones to facilitate the use of the data collection platform, and person-hours devoted to configuring devices and managing incoming data.” **The Conclusions jump straight into the recommended road map, but do not initially distil the key issues faced by the program, as revealed by the preceding analyses.**

*Thank you for highlighting this important issue. We have created a new table (table 4 [please find in the body of the manuscript submitted with this letter]) that lists the key issues faced by the program (as learned from the IVC-DISC contextual matrix) and distils how each issue is addressed in various phases, including their implementation considerations and potential barriers. To bridge this gap, we added the following to the Discussion section:* “We distill each key issue faced by the information system in **Table 4** and further describe how these are addressed by a multi-phase approach of system improvements.” *These issues are discussed in more detail in the corresponding ‘Phases’ of the Discussion section.* **Reviewer #2: see below
Reviewer #3: Yes
--------------------
Editorial and Data Presentation Modifications?
Use this section for editorial suggestions as well as relatively minor modifications of existing data that would enhance clarity. If the only modifications needed are minor and/or editorial, you may wish to recommend “Minor Revision” or “Accept”.
Reviewer #1: Some minor editorial points and some points of biological specificity that might be refined...
L38 "involves a freshwater copepod (tiny crustacean) in its lifecycle" is an oddly informal detail for the first line of the article - perhaps this detail might be framed more biologically and moved elsewhere in the introduction.**

*We have elected to delete some of the details of the Guinea worm lifecycle, in order to make room for additional context about public health informatics at the suggestion of other reviewers. (Otherwise the Introduction alone would have been over a thousand words long.) The detailed transmission cycle is not required to understand this paper, which is focused on information systems.* **L43 It has been a convention that the term "cases" refers only to humans, while animals are termed "infections". Some people (not me particularly) adhere with some passion to this distinction. Given the prominence of this change in vocabulary, in the early stages of the Introduction, the authors might want to reflect on whether this is a deliberate and considered change.**

*It is true that the CGWEP uses the term “cases” when referring to infections in humans and “infections” when referring to animals. Upon writing this manuscript we did have deliberate discussions about the use of this terminology, and ultimately decided that that “case” was most appropriate for referring to infections in all hosts. The audience for this manuscript is the broader scientific community, and outside of the CGWEP, there is typically no distinction between case/infection for humans/animals.* **L43 Perhaps the 2020 numbers might be used now that they are available.**

*Thank you for this suggestion. We have added provisional data for 2020.* **L51 Given its use to control crustaceans in this context, temephos is perhaps not best described as an insecticide. It is often described as an organophosphate larvicide or similar.**

*Yes, that is a good point. As previously mentioned, we ultimately decided to remove this statement. Since this manuscript does not mention Abate again, we do not feel it is essential information for the reader to understand the remainder of the paper.* **L52 More strictly, the filters yield water that is free of copepods that might carry Guinea worm larvae**

*Please see comment above.* **L57 I would not consider relational databases, or Excel / Access to be recent technological advances. Clearly there are some highly relevant advances that are relatively recent, such as Cloud-based systems. Just tweak the text a bit here. Some tools are only basic and it will come as a surprise that there is such a reliance on paper record keeping, given how long established basic tools are. Moreover, the paper advocates moving away from simple but inefficient systems like Excel spreadsheets.**

*Thank you for the suggestion, we made the changes as advised.* **L61 Slight hyperbole about a tipping point in history, perhaps?**

*Changed accordingly.* **L81 This line is contradictory, in that it identifies historical data collection practices *at the time* of the study. Which is it? Is this what the investigators found, or is it how they understood things were in the past? Perhaps this needs to be stated explicitly, by giving time frames?**

*As suggested in the comments above, these sentences were moved to the Introduction. We removed the contradiction from the sentence there as well. This now rewritten as:* “At the time of this investigation, Guinea worm surveillance data were collected in a siloed manner with inefficiencies in data management processes.” **L83 individual "host"? Is this asking for linkages between disparate information about the same host/worm to be linked, or for information about the host and the worm to be linked, or both?**

*Upon reflection, this really applies to ‘cases’ rather than individual hosts or worms specifically. Here too, the sentence was moved to the Introduction:* “For example, information collected regarding the same Guinea worm case was not linked because data were collected in isolation via paper-based surveys.” **L149 The Results start with some unnecessary recapitulation of the Methods.**

*We moved this to the last paragraph of the methods section, as a summary of the methods and to tie it with the Results section.* **L262 I think this is the first mention of NEMO/ELMO, which requires some explanation, as it then dominates the remaining recommendations.**

*Thank you for the suggestion. We added a brief description of NEMO/ELMO:* “NEMO/ELMO, an open-source and cloud-based mobile data collection and reporting system developed by The Carter Center, because the platform is familiar to field staff [35, 36].”, *with citations to for it.* **Reviewer #2: see below
Reviewer #3: None
--------------------
Summary and General Comments


Use this section to provide overall comments, discuss strengths/weaknesses of the study, novelty, significance, general execution and scholarship. You may also include additional comments for the author, including concerns about dual publication, research ethics, or publication ethics. If requesting major revision, please articulate the new experiments that are needed.
Reviewer #1: This is potentially a valuable account of the data and information operations of a program with global significance. It is of general interest to other disease control and eradication programs and, in principle, is within scope for the journal.
The account of information flow and challenges to effective use of data is pointed, clear and useful.
That said, in parts this manuscript reads rather more like a contract report than a research paper and the tone and structure needs some attention to bring it up to the latter standard. Broader context from the literature in this area is largely lacking.
There are some key issues that I think should be addressed before publication, that would greatly improve the value of the paper.
The Introduction is brief and highly specific, giving some basic details of the GWEP in Chad and of the basics of informatics analyses. However, there is scope to introduce more broadly the important role of informatics, and improvement therein, to disease eradication and control, including successful programs and those that are currently struggling. Some contrast between the specifics of public health informatics in challenging environments found in low to middle income countries, such as are found in the remaining GW endemic countries, compared to high income countries would be valuable.**

*We made significant changes throughout the paper in direct response to the reviewers’ comments (also addressed in detail in this letter) to improve the tone and structure for a research paper.*

*We have attempted to improve this work by adding a summary of related work to the introduction. Some previous informatics work related to NTDs added are:*

- *A viewpoint outlining the use of technology for NTDs [8]*
- *An assessment of paper-based and digital tools of data collection in low to middle income countries, specifically as it pertains to data quality [10]*
- *A pilot study comparing electronic data collection and paper-based questionnaires for trachoma cases in Ethiopia [11]*
- *A review of digital health technologies used in neglected tropical diseases [12]*
- *A report describing an SMS based tool for assessing lymphatic filariasis in Malawi and Ghana [13]*
- *A systematic review of innovative tools used by mobile health strategies to tackle skin NTDs [14]*

*Previous work specific to Guinea worm disease in Chad cited in the manuscript:*

- *Summary of eradication efforts – 2017-2018 and 2018-2019 [1, 16]*
- *Description of CGWEP surveillance system and analyses of the data from the surveillance system of CGWEP [4, 20, 23]*
- *Epidemiology of Guinea worm in Chad [5, 6]*

*Previous work related to Guinea worm eradication efforts cited in the manuscript:*

- *Cost-effectiveness [17]*
- *Challenges [18]*

*We changed the introduction section significantly and discussed the main findings of the studies published from low to middle income countries that reported on technology tools used for NTDs. The following text was added in the ‘Public Health Informatics Context’ subsection (of Introduction):* “Previous works comparing electronic data collection to paper-based data collection for NTDs have shown that data collected through electronic sources were less expensive, more efficient, and produced higher quality data with fewer errors [10, 11]. However, there are few studies describing digital health technologies in surveillance, management, and treatment of NTDSs [12]. Although some studies reported on enhancements of the surveillance and management of NTDs, they were limited to the description of specific tools used for those processes [12, 13, 14].”

*We summarized that a gap exists in describing a standardized approach to assess and modernize surveillance information systems; all related published studies in NTDs in low to middle income countries describe specific digital and mobile tools used for specific purposes:* “Previously published studies revealed a gap in a systems-level assessment of information systems unique to NTDs [10], and therefore, this approach could also be adopted by other NTD programs [21].” **While it is clear that the IVC-DISC approach allows some formal analysis of the informatics challenges, these are not set in context or analysed in any obvious way, that would then substantiate the roadmap towards improvement. While some of the informatics shortcomings/failings are clear (too many paper forms, clunky data handling, data not well connected, risk of data loss), it is not substantiated or made clear how great the benefit would be if this were fixed. It is not specifically addressed how such flawed systems have nevertheless worked so well up to this point, and/or how indispensable improvements are at this stage. Effectively, is this essential works at this point, or is it "nice to have"?**

*This is the first gap analysis conducted at CGWEP to inform areas of improvement needed in the surveillance information system. To clarify how the proposed phases in the roadmap would specifically address each of the issues we identified in the IVC-DISC framework, we added a table that outlines each phase, the issues addressed (i.e. the benefits of each phase), and the possible costs incurred during implementation (table 4 [please find in the body of the manuscript submitted with this letter]).*

*The reviewer brings up an interesting point about how the system worked previously, and why these improvements are needed now, which relates to a subsequent comment about why this is specifically relevant to an eradication program. There are two main points that we have added to the text to explain this better: First, that in the context of eradication, identifying and containing every single case of Guinea worm is important – and doing so becomes increasingly difficult as there are fewer numbers of cases. Second, at the same time, the number of “rumors” of Guinea worm has increased substantially (dog rumors in particular). The enormous increase in rumors over time has highlighted the need for improved information systems.*

*We added the following text to the Introduction to better explain this:* “Although the existing information system worked well in the past, as evidenced by the 99.9% reduction in human Guinea worm cases since the mid-1980s [1,15, 16], in the context of eradication, identifying and containing every single case becomes increasingly difficult as cases decline [17, 18]. At the same time, the number of Guinea worm “rumors” (unverified reports of possible cases) in Chad has increased substantially. For example, Guagliardo et al (2020) reported that there were 504 dog rumors in 2015 compared to 15,511 rumors in 2018 [4]. The enormous increase in rumors over time has underscored the need for improved information systems to facilitate detection, tracking, and containment.” **Proposals, e.g. for a data governance plan, are introduced with little substantiation of options beyond "contractor-type" recommendations. These recommendations then stop short of explicit content, requirements and implications. These could perhaps be tabulated to be efficient. This section is particularly suggestive of a contractor report, as opposed to a research paper. However, it again stops short of spelling out the implications in terms of cost, scale, timelines for the recommended roadmap, and the context of a plan for disease elimination in Chad that currently measures out over only a few years.**

*Thank you for this valuable feedback. The goal of this submitted manuscript was to report an approach to assess a surveillance information system and methods to make improvements on the strengths and weaknesses identified in that process. More importantly, we intend to demonstrate a replicable and reproducible approach to improve surveillance information systems. Keeping this in mind, we have created a new table listing the key issues identified and further describe what resources and potential barriers such system improvements may face (Table 4). This should substantiate how each key issue can be improved and/or solved with the proposed phases and specific interventions within those phases. We also estimate resources, requirements, and barriers for each system improvement. We have acknowledged that actual cost and timeline will vary on various local factors and context.*

*Additionally, we further acknowledge this in the ‘Limitations’ subsection by adding the following text:* “The phased plan we propose here may not be suitable for all NTD programs because of the financial and human resources required and because of connectivity issues in remote areas that may hinder the ability to transmit and receive data. In the process of this transition CGWEP did incur costs related to monthly data transfer and storage, the cost of purchasing 700 cell phones to facilitate the use of the data collection platform, and person-hours devoted to configuring devices and managing incoming data.” **Finally, and perhaps most importantly, the paper misses an absolutely key point in relation to the specificities of *eradication* and of a disease with human and animal hosts, as opposed to effective control. To identify the importance of informatics to eradication, and certification, is a key requirement of the Guinea worm program, that generalises to other eradication programs, and yet this specificity is not mentioned at all anywhere in the paper. Neither does the paper highlight the specific challenge facing Guinea worm eradication of the involvement of people and animals, and any informatics challenges this generates.**

*We thank the reviewer for bringing up this important point. As we mentioned previously in the response to the comment above, we have added the following text to the Introduction:* “Although the existing information system worked well in the past, as evidenced by the 99.9% reduction in human Guinea worm cases since the mid-1980s [1,15, 16], in the context of eradication, identifying and containing every single case becomes increasingly difficult as cases decline [17, 18]. At the same time, the number of Guinea worm “rumors” (unverified reports of possible cases) in Chad has increased substantially. For example, Guagliardo et al (2020) reported that there were 504 dog rumors in 2015 compared to 15,511 rumors in 2018 [4]. The enormous increase in rumors over time has underscored the need for improved information systems to facilitate detection, tracking, and containment.”

*We have also added a sentence describing the challenge of dog infections:* “The discovery of canine infections in dogs in Chad has posed a significant challenge to eradication efforts [4,5].”

*To further describe the generalizability our phased approach to other NTD programs, we have added:*

- *In ‘Phase I’ of the Discussion section:*
  - “A data governance plan also promotes institutional memory and shortens the learning curve for incoming staff. Importantly, this plan does not require intensive resource allocation for development or implementation, and ultimately saves both time and resources [34]. Therefore, this Phase in the roadmap could easily be adapted to any disease control, elimination, or eradication program that relies on surveillance data.”
- *In ‘Phase II’ of the Discussion section, we cite another NTD program that has utilized an electronic data entry and centralized storge systems via a mobile application-based data collection platform and added the following text:*
  - “Resources required to implement this phase include (but are not limited to) purchase of centralized storage system/service and staff dedicated to configuring and maintaining this database. Some NTD programs already use electronic data entry and centralized storage systems. For example, the Mission Rabies App has been used to collect information about dog vaccinations, rabies community education, and field surveys in 16 countries [37].”
- *In ‘Phase III’ of the Discussion section:*
  - “Additional resources for electronic data collection would include the purchase of electronic devices (such as mobile phones) and staff dedicated to programming the devices.”
  - “This bidirectional information exchange, however, would require significant technology and human resources to manage the system, which may not be available to many NTD programs.”
- *In ‘Phase IV’ of the Discussion section, we cite an example of how integrating NTD data onto a cloud platform provided dynamic analyses and visualization capability and added the following text:*
  - “The NTD Mapping Tool, for example, also provides dynamic analyses and visualizations by integrating NTD data onto a cloud platform and reduces the burden for reporting and provides a tool for planning, implementation, and evaluation of NTD control activities [38].”

**Reviewer #2: This paper talks about an effort of assessing the Chad Guinea Worm Surveillance Information System. The authors used Information Value Cycle and Data Information System-Context framework to understand the information generated and identify the gaps.
As the paper stated, this topic is very important and the outcome of the information system has great social impacts, so this is very important work. However, since this manuscript is marked as a research paper, my review will focus on the innovation of the research questions and the validity of the research method.
Firstly, there is no literature of the related work. There probably only one Guinea Worm Surveillance Information System in Chad, but there must be related work on examining the validity and the gap of an information system for diseases. Consequently, there should be a related work section to indicate the limitations of our scientific understanding of the problems, and the innovation of this paper.**

*Thank you for highlighting this concern. As summarized in the response to the comments above, we have discussed many other studies relevant to informatics work in NTDs, CGWEP surveillance, and Guinea worm eradication.*

*Some previous informatics work related to NTDs added are:*

- *A viewpoint outlining the use of technology for NTDs [8]*
- *An assessment of paper-based and digital tools of data collection in low to middle income countries, specifically as it pertains to data quality [10]*
- *A pilot study comparing electronic data collection and paper-based questionnaires for trachoma cases in Ethiopia [11]*
- *A review of digital health technologies used in neglected tropical diseases [12]*
- *A report describing an SMS based tool for assessing lymphatic filariasis in Malawi and Ghana [13]*
- *A systematic review of innovative tools used by mobile health strategies to tackle skin NTDs [14]*

*Previous work specific to Guinea worm disease in Chad cited in the manuscript:*

- *Summary of eradication efforts – 2017-2018 and 2018-2019 [1, 16]*
- *Description of CGWEP surveillance system and analyses of the data from the surveillance system of CGWEP [4, 20, 23]*
- *Epidemiology of Guinea worm in Chad [5, 6]*

*Previous work related to Guinea worm eradication efforts cited in the manuscript:*

- *Cost-effectiveness [17]*
- *Challenges [18]*

*We went on to highlight he gaps in this area and how we hope to fill that:*

- “Previous works comparing electronic data collection to paper-based data collection for NTDs have shown that data collected through electronic sources were less expensive, more efficient, and produced higher quality data with fewer errors [10, 11]. However, there are few studies describing digital health technologies in surveillance, management, and treatment of NTDSs [12]. Although some studies reported on enhancements of the surveillance and management of NTDs, they were limited to the description of specific tools used for those processes [12, 13, 14].”
- “Although a previous, rigorous surveillance evaluation had been conducted [20], it was determined that an additional assessment would be needed specifically to address informatics needs.”
- “Previously published studies revealed a gap in a systems-level assessment of information systems unique to NTDs [10], and therefore, this approach could also be adopted by other NTD programs [21].”

**Secondly, there are no research questions presented in the paper. Consequently, it is not clear the focus of this paper, and the set of specific issues or questions that the authors want to address in this paper. As a result, it is not clear whether or not the results that the authors presented in this paper are the important ones and/or the comprehensive list of ones.**

*As we note above, we acknowledge that this is an unconventional paper. However, there are limited informatics or data management framework-related papers and this manuscript attempts to fill that gap in literature. While we don’t propose a specific hypothesis, thank you for raising your concern regarding adding more clarity on the objective of the study. This is the first gap analysis conducted at CGWEP to inform areas of improvement needed in the surveillance information system. We added context for the objective of this study, based on the work to be done to assess and improve CGWEP information system:* “At the time of this investigation, Guinea worm surveillance data were collected in a siloed manner with inefficiencies in data management processes. For example, information collected regarding the same Guinea worm case was not linked because data were collected in isolation via paper-based surveys. Although the existing information system worked well in the past, as evidenced by the 99.9% reduction in human Guinea worm cases since the mid-1980s [1,15, 16], in the context of eradication, identifying and containing every single case becomes increasingly difficult as cases decline [17, 18]. At the same time, the number of Guinea worm “rumors” (unverified reports of possible cases) in Chad has increased substantially. For example, Guagliardo et al (2020) reported that there were 504 dog rumors in 2015 compared to 15,511 rumors in 2018 [4]. The enormous increase in rumors over time has underscored the need for improved information systems to facilitate detection, tracking, and containment.”

*Specific to CGWEP, there were previous studies on the surveillance system, but no previous work to examine the information system supporting the surveillance system*: “Although a previous, rigorous surveillance evaluation had been conducted [20], it was determined that an additional assessment would be needed specifically to address informatics needs.”

*An extensive search of published related studies revealed a major gap for this work. Prior studies reported on specific tools used in NTDs, none described a reproducible approach for any systemic assessment and improvement of the information system. We explain this in the manuscript in the following text: “*Previous works comparing electronic data collection to paper-based data collection for NTDs have shown that data collected through electronic sources were less expensive, more efficient, and produced higher quality data with fewer errors [10, 11]. However, there are few studies describing digital health technologies in surveillance, management, and treatment of NTDSs [12]. Although some studies reported on enhancements of the surveillance and management of NTDs, they were limited to the description of specific tools used for those processes [12, 13, 14].”

*With the context for this study added above, we emphasize the goal of this study:* “The purpose of this report is to describe the approach and methods used to in the Info-Aid, and to outline a roadmap to improve CGWEP information systems’ data flow, upstream and downstream data management practices, and system inefficiencies. Previously published studies revealed a gap in a systems-level assessment of information systems unique to NTDs [10], and therefore, this approach could also be adopted by other NTD programs [21].” **Thirdly, there is a brief introduction of the two mental models used in this paper, and a reference is given for the two models, which is a book chapter. However, there is no further justification on the literature around these two models. Are they well known models in the field? Have other related work used these two models and obtained insight results? Yes, these two models helps to identify the functions and structure of the system, but why are these two aspects important for understanding the information generated and the gap that the authors seek? Particularly, the gap can exist at any part of the information system, so how do these two models help to identify the gap?**

*We added references upon which the IVC was developed [26, 27]. We did the same for DISC [30, 31, 32]. We also added references of other projects that have used the IVC-DISC framework [28, 29]. Beyond the explanation that were present in the earlier version of the submitted manuscript, we further added and modified the following text in the manuscript:*

- “. The IVC, initially adapted from an early publication by Taylor et al, addresses the *function* of the information system [27] and the DISC deals with its *structure*; the combined approach provides a more holistic diagnosis of the entire information system in its current state and better elucidates system gaps.”
- “The DISC framework recognizes the significance of the different personal, organizational, and environmental contexts that contribute to the success of an information system [30, 31, 32].”
- “This IVC-DISC framework has been frequently used to guide the solutions for various informatics problems in public health [28, 29].”

*We further added explanation in the last paragraph of the ‘Mental Models’ subsection of the ‘Methods’ section to highlight why we chose this model: “*We mapped the concepts identified in the IVC to the DISC domains to create a contextual analysis matrix. This IVC-DISC framework has been frequently used to guide the solutions for various informatics problems in public health [28, 29]. Concepts identified using the IVC were further categorized into granular contexts using the DISC framework. This IVC-DISC contextual analysis matrix reveals each concept identified in the IVC in the context of data, technology, people and process, organization, and environment. It is an evaluation model for assessing information systems, the value that they create, and their data flow. We used IVC-DISC framework as it breaks down contributors to the information system that are otherwise difficult to isolate. This allows for a holistic examination of these contributors and at the same time also allows for a measurable and quantifiable elicitation of these contributors and their roles in the system as a whole.”

**The four phases presented in the discussion section sound very important, however, it is not clear to me how these phases are mapped to the results presented in the result section.**

*Thank you for this feedback. This comment is similar to comment above and is addressed there, please see above.* **Overall, this is a very important project report, but it is not a high quality research paper to be published on a PLOS journal yet.**

*We acknowledge that this work is unconventional. Informatics challenges are common in the NTD space, and we felt that it could be beneficial to others to read about the approach we used here to improve information systems.* **Reviewer #3: This is a rather unconventional submission for PLOS NTDs, as it does not contain any research, data or data analysis. The manuscript reports an analysis of the current data recording procedures used by the Chad Guinea Worm eradication program (CGWEP), and provides recommendations for how the program might improve the speed, accuracy and efficiency of their data collection and analysis activities. The CGWEP collects data of provisional and confirmed cases using four different forms, each of which contains a large number of questions that need to be completed. The authors conducted an analysis of the forms and the data collection and analysis process using a combination of two mental model frameworks and identified a number of redundancies and potential weak points in the current process. Based upon this, they proposed a four-step process to allow the CGWEP to move to a cloud-based data capture and analysis platform.**

*We acknowledge that this is an unconventional submission for PLoS NTDs, but would argue that there are past examples of other publications that do not contain “data” in the conventional sense. Case reports, for example, contain valuable clinical information about presentation of disease in humans. The journal’s scope also includes surveillance of NTDs, and informatics is an important component of surveillance. We would also suggest that the work presented here is in fact an analysis, although admittedly not quantitative in nature.*  **1. The authors propose a gradual process of migrating the current paper-based system to a cloud based central database. While it is obvious that this will increase the timeliness of data reporting, reduce data entry and transcription errors and will result in a central repository for all data, the authors do not provide any estimate of the cost of the cloud conversion. How many data entry devices (cell phones tablets, etc) will be needed to effect this conversion? Will this be affordable? How daunting are the connectivity issues for data upload in Chad likely to be?**

*This is an important point, and we thank the reviewer for bringing this to our attention. Regarding the estimated costs associated with this process, we estimate the monthly costs for data transfer/storage was about $300, in addition to the cost of 700 phones (~$200/phone) to use ODK Collect. We acknowledge that some NTD programs are underfunded and that these costs alone are a hindrance to implementing our proposed plan. The connectivity issue in Chad has been manageable to date. The devices are storing the data collection and they upload almost every day either via wifi or cellular data. Also, we are exploring the use of SMS for really remote locations since NEMO allows for three types of submission (web form, ODK Collect, and SMS). As far as person hours, IT staff based in Atlanta configure all of the devices, so they are ready to go when they arrive in Chad. We did add a data analyst to the GW team to help wrangle all of the data coming in, but this person is not devoted solely to Chad. (About 60 % of his time is dealing with the Chad data.)*

*We have added the following text to the Limitations of the Discussion:* “The phased plan we propose here may not be suitable for all NTD programs because of the financial and human resources required and because of connectivity issues in remote areas that may hinder the ability to transmit and receive data. In the process of this transition CGWEP did incur costs related to monthly data transfer and storage, the cost of purchasing 700 cell phones to facilitate the use of the data collection platform, and person-hours devoted to configuring devices and managing incoming data.”

*Some of the new work we are doing with ODK Collect will eventually result in providing longitudinal data on the device. The GW team is working directly with ODK on the design of software enhancements. This is an additional investment, but one that hopefully will make it easier to track dog cases. Relatedly, we are also working on ways to catalog the dogs in a national registry that eventually could inform field staff if they identify a dog that migrates from one village to another.* **2. The authors suggest that this process might be a useful guide for other disease elimination programs to follow to move their data systems to the cloud as well. I agree that this may be very useful. But most of the NTD elimination programs are not nearly as advanced as the Guinea Worm program, and as such, they employ many more people. For example, the river blindness elimination programs in Africa all rely upon volunteer community drug distributors (CDDs) to distribute the needed drugs and to keep the records of who has been treated and who has not. Each community has a number of CDDs – usually at least one per kinship group. Currently, all the treatment records are kept by th CDs on paper. It would seem to me to be very expensive to try to migrate the CDD collected data to a cloud-based platform. I would like to see the authors expand further on the generality of what they have done here to other PC NTDs.**

*We recognize that such informatics interventions and systems improvements will require some resources. All local and program-specific implementation of this approach described in this manuscript will need to have some program-specific considerations. We have acknowledged this as a limitation in our Discussion section:* “The phased plan we propose here may not be suitable for all NTD programs because of the financial and human resources required and because of connectivity issues in remote areas that may hinder the ability to transmit and receive data. In the process of this transition CGWEP did incur costs related to monthly data transfer and storage, the cost of purchasing 700 cell phones to facilitate the use of the data collection platform, and person-hours devoted to configuring devices and managing incoming data.”

*We added Table 4 to help generalize (and make more evident) how key issues identified during the assessment was addressed in our study. We also acknowledge that actual expense will be dependent on various local factors and context, we layout the resources needed for implementation of each phase. To further describe the generalizability our phased approach to other NTD programs, we have added:*

- *In ‘Phase I’ of the Discussion section:*
  - “A data governance plan also promotes institutional memory and shortens the learning curve for incoming staff. Importantly, this plan does not require intensive resource allocation for development or implementation, and ultimately saves both time and resources [34]. Therefore, this Phase in the roadmap could easily be adapted to any disease control, elimination, or eradication program that relies on surveillance data.”
- *In ‘Phase II’ of the Discussion section, we cite another NTD program that has utilized an electronic data entry and centralized storge systems via a mobile application-based data collection platform and added the following text:*
  - “Resources required to implement this phase include (but are not limited to) purchase of centralized storage system/service and staff dedicated to configuring and maintaining this database. Some NTD programs already use electronic data entry and centralized storage systems. For example, the Mission Rabies App has been used to collect information about dog vaccinations, rabies community education, and field surveys in 16 countries [37].”
- *In ‘Phase III’ of the Discussion section:*
  - “Additional resources for electronic data collection would include the purchase of electronic devices (such as mobile phones) and staff dedicated to programming the devices.”
  - “This bidirectional information exchange, however, would require significant technology and human resources to manage the system, which may not be available to many NTD programs.”
- *In ‘Phase IV’ of the Discussion section, we cite an example of how integrating NTD data onto a cloud platform provided dynamic analyses and visualization capability and added the following text:*
  - “The NTD Mapping Tool, for example, also provides dynamic analyses and visualizations by integrating NTD data onto a cloud platform and reduces the burden for reporting and provides a tool for planning, implementation, and evaluation of NTD control activities [38].”

**3. One suggestion that the authors had concerning the advantage of an electronic data entry platform was that the platform could be programmed to auto fill much of the data on a given individual, saving the person entering the data time. But in the case of the CGWEP, the number of cases that are being reported is quite small, as the infection is near elimination. Given the relative rarity of the infection, how often does the program field case reports from the same individual more than once?**

*Autofill will not necessarily help with multiple infections. Rather, it will help with data quality and completeness on the same individual since there will be multiple forms collecting information regarding different things (e.g., rumor form, surveillance questionnaire).*

*While the number of reported human rumors and confirmed cases are decreasing and are relatively low, the number of rumors in dogs has increased exponentially. This new development potentially can be tackled with electronic data entry with ‘autofill’ functionality. We have added this to the manuscript:* “At the time of this investigation, Guinea worm surveillance data were collected in a siloed manner with inefficiencies in data management processes. For example, information collected regarding the same Guinea worm case was not linked because data were collected in isolation via paper-based surveys. Although the existing information system worked well in the past, as evidenced by the 99.9% reduction in human Guinea worm cases since the mid-1980s [1,15, 16], in the context of eradication, identifying and containing every single case becomes increasingly difficult as cases decline [17, 18]. At the same time, the number of Guinea worm “rumors” (unverified reports of possible cases) in Chad has increased substantially. For example, Guagliardo et al (2020) reported that there were 504 dog rumors in 2015 compared to 15,511 rumors in 2018 [4]. The enormous increase in rumors over time has underscored the need for improved information systems to facilitate detection, tracking, and containment.”

***Additional Changes (Outside of Reviewer Requests)***

*We have elected to cut some text from the Introduction, in order to make room for the additional information that the reviewers requested. (For example, some of the biological details of the Guinea worm life cycle.)*

*Similarly, we have cut some of the text in the Discussion to make room for reviewer requests.*
